# Significant Depletion of CD4^+^ T Cells Occurs in the Oral Mucosa during Simian Immunodeficiency Virus Infection with the Infected CD4^+^ T Cell Reservoir Continuing to Persist in the Oral Mucosa during Antiretroviral Therapy

**DOI:** 10.1155/2015/673815

**Published:** 2015-04-30

**Authors:** Jeffy George, Wendeline Wagner, Mark G. Lewis, Joseph J. Mattapallil

**Affiliations:** ^1^Uniformed Services University of the Health Sciences, Bethesda, MD 20814, USA; ^2^Bioqual Inc., Rockville, MD 20850, USA

## Abstract

Human and simian immunodeficiency virus (HIV and SIV) infections are characterized by manifestation of numerous opportunistic infections and inflammatory conditions in the oral mucosa. The loss of CD4^+^ T cells that play a critical role in maintaining mucosal immunity likely contributes to this process. Here we show that CD4^+^ T cells constitute a minor population of T cells in the oral mucosa and display a predominantly central memory phenotype mirroring other mucosal sites such as the rectal mucosa. Chronic SIV infection was associated with a near total depletion of CD4^+^ T cells in the oral mucosa that appear to repopulate during antiretroviral therapy (ART). Repopulating CD4^+^ T cells harbored a large fraction of Th17 cells suggesting that ART potentially reconstitutes oral mucosal immunity. However, a minor fraction of repopulating CD4^+^ T cells harbored SIV DNA suggesting that the viral reservoir continues to persist in the oral mucosa during ART. Therapeutic approaches aimed at obtaining sustainable CD4^+^ T cell repopulation in combination with strategies that can eradicate the latent viral reservoir in the oral mucosa are essential for better oral health and long-term outcome in HIV infected patients.

## 1. Introduction

Oral mucosa is an active site for the onset of numerous opportunistic infections such as oral candidiasis, Epstein Barr virus (EBV) associated oral hairy leukoplakia, Kaposi sarcoma, periodontitis, and other ulcerative lesions in HIV infected patients [1–8]. The loss of mucosal immunity in the oral mucosa is generally thought to contribute to this process. CD4^+^ T cells play a key role in oral mucosal immunity and numerous studies have documented the loss of CD4^+^ T cells in the gastrointestinal tract (GIT) such as the small and large intestinal mucosa during HIV and SIV infections [9–14]. The advent of highly active ART (HAART) has led to a decrease in the incidence of oral infections in some groups of patients suggesting that HAART likely restores some of the mucosal immunity that is lost during HIV infection. It is not clear if the perturbations of CD4^+^ T cells seen in the lower GIT during HIV infection occur to a similar extent in the oral mucosa and if HAART effectively restores CD4^+^ T cells in the oral mucosa.

CD4^+^ T lymphocytes play an important role in the generation and maintenance of both humoral and cellular immune responses by providing T cell help. Unlike peripheral tissues, the CD4^+^ T cells in the GIT display a predominantly memory phenotype [12]; these cells are critical for the generation of secondary immune responses to the previously exposed pathogens in these tissues. Though numerous studies have evaluated peripheral CD4^+^ T cell counts and associated them with onset of opportunistic infections, few studies have examined the dynamics of CD4^+^ T cells in oral mucosa during HIV infection and HAART. CD4^+^ T cells in the oral mucosa, as in other mucosal tissues, are probably highly susceptible to HIV infection and their loss likely compromises the integrity of oral immune system.

We sought to characterize the nature and phenotype of CD4^+^ T cells in the oral mucosa and determine if CD4^+^ T cell dynamics were altered during HIV infection and antiretroviral therapy (ART) using the SIV infected nonhuman primate model. Rhesus macaques infected with SIV has been a valuable model to study HIV pathogenesis, and studies have shown SIV infected macaques display similar oral pathologies and susceptibilities to opportunistic infections such as EBV and candidiasis as seen in HIV infected subjects [15–17].

Our results show that the prevalence and phenotype of CD4^+^ T cells in the oral mucosa mirror those of CD4^+^ T cells in the rectal mucosa, with a repopulation of CD4^+^ T cells during ART. Interestingly, a fraction of CD4^+^ T cells repopulating the oral mucosa were found to harbor SIV DNA suggesting that the infected viral reservoir continues to persist in oral mucosa during therapy.

## 2. Materials and Methods

### 2.1. Animals, Infection, and Samples

Archived samples from rhesus macaques (*Macaca mulatta*) of Indian origin were used for this study. The animals were housed in accordance with the American Association for Accreditation of Laboratory Animal guidelines and were seronegative for SIV, simian retrovirus (SRV), and simian T cell leukemia virus (STLV) type 1. Animals were infected intravenously with ~1000 TCID50 of uncloned pathogenic SIVmac251.

Peripheral blood and oral and rectal mucosal samples were obtained from five healthy animals, eight animals chronically infected with SIV, and four animals that were treated with antiretroviral drugs PMPA (Tenofovir) and FTC (Emtricitabine) continuously for more than 6 months until sacrifice.

PBMC was isolated by density gradient centrifugation and mucosal samples were processed by enzymatic digestion followed by enrichment over a percoll gradient as described previously [10, 18]. Plasma viral loads were determined by real-time PCR with an ABI Prism 7700 sequence detection system (Applied Biosystems) using reverse-transcribed viral RNA as template using methods previously described [19].

### 2.2. Antibodies and Flow Cytometry

Isolated cells were labeled with a combination of CD3-Cy7APC, CD4-PB, CD28-Cy5-PE, CD95-FITC, and CD8-Alexafluor700 and analyzed by flow cytometry using procedures described previously [10, 12, 18, 20–22]. All the antibodies were obtained from BD Biosciences (San Diego, CA) and titrated using rhesus macaque PBMC. Labeled cells were fixed with 0.5% paraformaldehyde and analyzed using a Becton Dickinson LSR II flow cytometer. For analysis of SIV-gag DNA in CD4^+^ T cells, cells were labeled as above and CD4^+^ T cells were sorted using a BD FACS Aria sorter.

IL-17 production in CD4^+^ T cells was determined after short-term stimulation with 10 ng/mL of PMA (phorbol myristate acetate; Sigma-Aldrich, Saint Louis, MO) and 500 ng/mL of Ionomycin (Sigma-Aldrich, Saint Louis, MO) in the presence of Brefeldin-A (BD Biosciences) for 4 hours. After stimulation, cells were labeled with anti-CD3-Cy7APC, CD8-Alexa-700, and CD4-PE. Cells were fixed in Cytofix/perm buffer (BD Biosciences) and labeled with anti-IL-17-APC (e-Biosciences). Labeled cells were fixed in 0.5% paraformaldehyde and analyzed using a Becton Dickinson LSR II flow cytometer.

### 2.3. qPCR Assay for SIV DNA in Sorted CD4^+^ T Cells

SIV-gag DNA in CD4^+^ T cells was determined using a highly quantitative PCR assay for SIV-gag as previously described [12] using SIV-gag specific primers and probes [23]. The assay was calibrated using a cell line that carried only a single copy of proviral SIV DNA [12]. Processed samples were analyzed using an ABI 7500 Taqman PCR instrument (Applied Biosystems Inc.).

### 2.4. Data Analysis

Flow cytometric data were analyzed using FlowJo version 9.2 (Tree Star, Inc., Ashland, OR). Statistical analysis was performed using *t*-test with GraphPad Prism Version 4.0 software (GraphPad Prism Software, Inc., San Diego, CA). A *p* < 0.05 was considered significant. Error bars represent standard error.

## 3. Results

### 3.1. CD4^+^ T Cells Are a Minor Population in the Oral Mucosa as Compared to Peripheral Blood and Have a Predominantly Central Memory Phenotype

**Figure 1:**
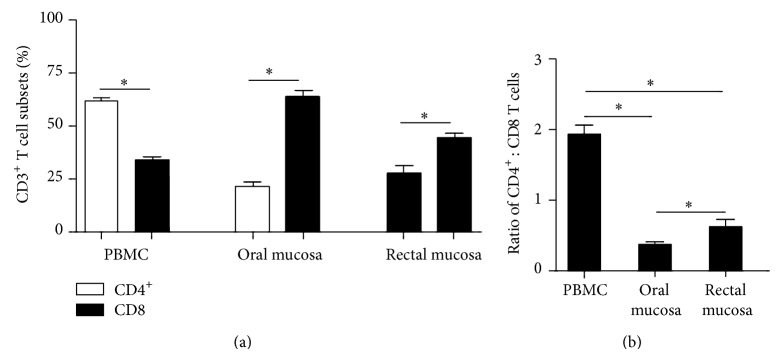
CD4^+^ T cells are a minor population of T cells in the oral mucosa of healthy animals. (a) The proportions of CD3^+^CD4^+^ and CD3^+^CD8^+^ T cells in peripheral blood and oral and rectal mucosa of healthy rhesus macaques. (b) The ratio of CD3^+^CD4^+^ : CD3^+^CD8^+^ T cells in peripheral blood and oral and rectal mucosa of healthy rhesus macaques.

We first examined the prevalence of CD4^+^ T cells in the oral mucosa of healthy animals to determine if they differed from that of other tissues such as the rectal mucosa and peripheral blood (Figure 1(a)). Peripheral blood was found to have a higher proportion of CD4^+^ T cells as compared to CD8 T cells; ratio of CD4^+^ T cells to CD8 T cells was ~2 : 1 (Figure 1(b)). In contrast, CD4^+^ T cells constituted a minor population of T cells in the oral mucosa with a majority of T cells being CD8 T cells. As such the ratio of CD4^+^ T cells to CD8 T cells was significantly inverted (<0.5) as compared to peripheral blood and resembled that of the rectal mucosa.

**Figure 2:**
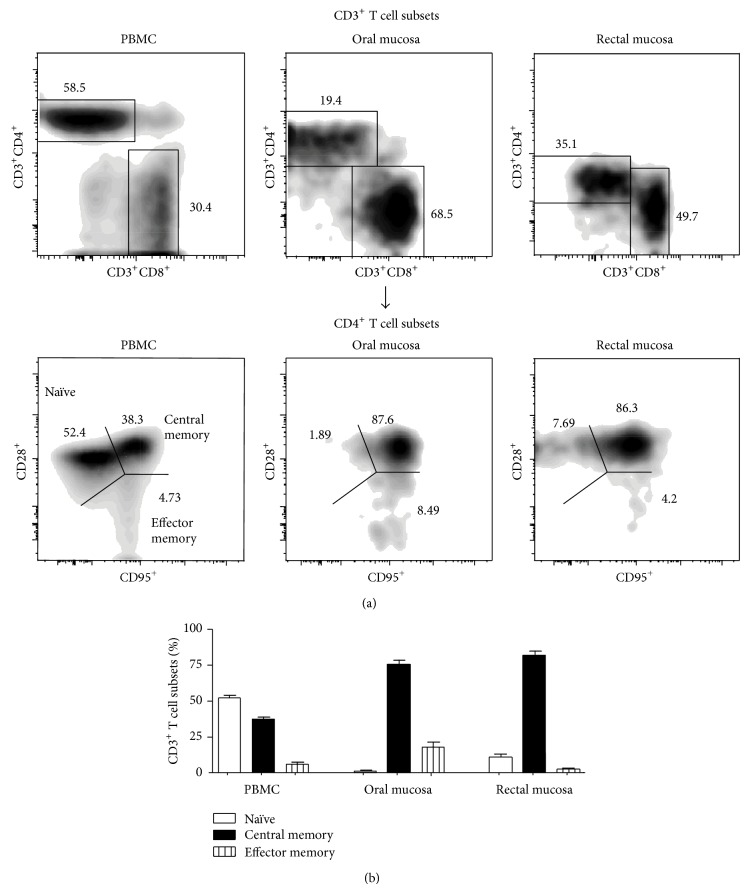
CD4^+^ T cells in the oral mucosa of healthy animals display a predominantly central memory phenotype. (a) Representative density plots showing the expression of CD28 and CD95 on CD3^+^CD4^+^ T cells from peripheral blood and oral and rectal mucosa. (b) Proportions of naïve, central memory, and effector memory CD4^+^ T cell subsets in peripheral blood and oral and rectal mucosa.

Previous studies have shown that mucosal CD4^+^ T cells displayed a predominantly memory T cell phenotype [10, 12, 22, 24, 25]. To determine if phenotype of CD4^+^ T cells in the oral mucosa differed from those of the rectal mucosa and periphery, we examined the expression patterns of CD28 and CD95 on CD4^+^ T cells from the oral mucosa of healthy rhesus macaques and compared them to CD4^+^ T cells in the rectal mucosa and peripheral blood (Figures 2(a)-2(b)). Naïve and memory CD4^+^ T cells were delineated based on the differential expression of CD28 and CD95 with memory CD4^+^ T cells expressing high levels of CD95 as compared to little or no expression of CD95 on naïve CD4^+^ T cells [10, 12, 21, 22, 26].

Our results showed that naïve CD4^+^ T cells were the predominant CD4^+^ T cell subset in peripheral blood as has been previously reported [12]. In contrast to peripheral blood, however, CD4^+^ T cells in the oral mucosa expressed a predominantly central memory T cell phenotype with most CD4^+^ T cells coexpressing CD28 and CD95. The phenotype of CD4^+^ T cells in the oral mucosa resembled that of CD4^+^ T cells found in the rectal mucosa.

### 3.2. Chronic SIV Infection Is Associated with a Near Total Depletion of CD4^+^ T Cells in the Oral Mucosa

We next examined the prevalence of CD4^+^ T cells in the oral mucosa of rhesus macaques that were chronically infected with SIV and compared them to rectal mucosa and peripheral blood CD4^+^ T cells.

**Figure 3:**
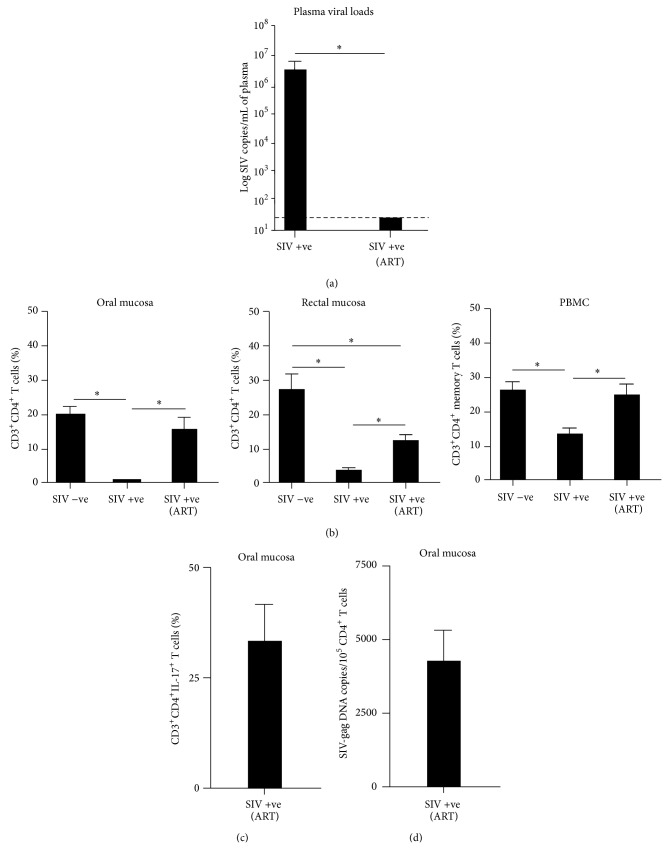
CD4^+^ T cells are significantly depleted in the oral mucosa during chronic SIV infection but repopulate during ART. (a) Plasma viral loads (limit of detection is 30 copies/mL of plasma) in untreated and treated animals. (b) Proportions of total CD4^+^ T cells in the oral and rectal mucosa and central memory CD4^+^ T cell subsets in peripheral blood during chronic SIV infection and ART. (c) Proportions of CD4^+^IL-17^+^ T cells in the oral mucosa during ART. (d) Level of SIV infection in sorted CD4^+^ T cells from the oral mucosa during ART.

SIV infected animals had ~6 logs of plasma viremia (Figure 3(a)), and chronic SIV infection was associated with a significant depletion of CD4^+^ T cells in both the oral and rectal mucosa (Figure 3(b)). As peripheral blood harbors a heterogeneous mix of naïve and memory CD4^+^ T cells, and memory CD4^+^ T cells like their counterparts in the oral and rectal mucosa are the primary targets for infection [12], we examined the dynamics of central memory CD4^+^ T cells in peripheral blood during chronic SIV infection and compared them to oral and rectal mucosal CD4^+^ T cells. We have previously shown that memory CD4^+^ T cells in peripheral blood and lymph nodes were significantly depleted to a similar extent as CD4^+^ T cells in the jejunum of SIV infected animals [12]. In line with these studies, chronic SIV infection was associated with a significant loss of memory CD4^+^ T cells in peripheral blood.

To examine if ART was accompanied by a repopulation of CD4^+^ T cells in the oral mucosa, we determined the prevalence of CD4^+^ T cells in the oral mucosa during ART and compared them to the rectal mucosa and peripheral blood. ART was associated with a significant suppression of plasma viral loads to levels that were below the limit detection (Figure 3(a)). CD4^+^ T cells were found to significantly repopulate the oral and rectal mucosa though the level of repopulation in the rectal mucosa was significantly lower than levels seen in healthy animals (Figure 3(b)). Peripheral blood central memory CD4^+^ T cells were found to significantly rebound after ART.

### 3.3. CD4^+^ T Cells Repopulating the Oral Mucosa during ART Harbor Th17 Cells

As T-helper-17 (Th17) cells play an important role in oral mucosal immunity [20, 27], we examined the expression of IL-17 in CD4^+^ T cells repopulating the oral mucosa during ART using intracellular staining and flow cytometry. Our results showed that ART was associated with restoration of Th17 cells with ~40% of CD4^+^ T cells in the oral mucosa expressing IL-17 suggesting that ART was capable of restoring oral mucosal immunity.

### 3.4. Infected Viral Reservoir Persists in CD4^+^ T Cells Repopulating the Oral Mucosa after ART

To determine if CD4^+^ T cells repopulating the oral mucosa harbored infected cells, we examined the level of SIV DNA in sorted CD4^+^ T cells from the oral mucosa of SIV infected animals during ART (Figure 3(d)). CD4^+^ T cells repopulating the oral mucosa carried ~4000 copies of SIV DNA/10^5^ CD4^+^ T cells suggesting that low levels of the SIV infected latent viral reservoir persisted in the oral mucosa during therapy even after complete suppression of plasma viremia. We were unable to sort CD4^+^ T cells from infected untreated animals due to the near total depletion of CD4^+^ T cells during chronic SIV infection.

## 4. Discussion

The oral mucosal environment is home to numerous opportunistic pathogens that remain under tight homeostatic control of the immune system. This control breaks down during HIV infection leading to the onset of various opportunistic infections and inflammatory conditions. CD4^+^ T cells play a central role in maintaining mucosal immune homeostasis and their loss in the gastrointestinal mucosa has been extensively documented. Surprisingly, however, limited studies have examined in detail either the loss or repopulation of CD4^+^ T cells during infection and therapy or characterized the nature of CD4^+^ T cells resident in the oral mucosa relative to other mucosal tissues. Our studies provide evidence that oral mucosa resembles other mucosal tissues such as the rectal mucosa as compared to peripheral blood; both oral and rectal mucosa had an inverted CD4 : CD8 ratio as compared to peripheral blood (Figure 1(b)). Interestingly, however, the oral mucosa was found to have significantly fewer CD4^+^ T cells than the rectal mucosa. Like the CD4^+^ T cells in the rectal mucosa, CD4^+^ T cells in the oral mucosa of healthy animals had a predominantly central memory phenotype with few naïve CD4^+^ T cells (Figures 2(a)-2(b)) suggesting that akin to the rectal mucosa the oral mucosa likely serves as an effector lymphoid compartment.

SIV infection was accompanied by a near total loss of CD4^+^ T cells in the oral mucosa that mirrored their loss in the rectal mucosa suggesting that oral mucosal immunity is significantly compromised as in the GIT during chronic stages of infection. Numerous studies have documented the loss of CD4^+^ T cells in mucosal tissues during HIV and SIV infections [9, 10, 12–14, 24, 25]. Continuous ART was accompanied by a repopulation of CD4^+^ T cells in both the oral and rectal mucosa though the extent of repopulation in the rectal mucosa was significantly lower than the proportions of CD4^+^ T cells prevalent in the rectal mucosa of healthy animals. As we did not sample tissues longitudinally, it is difficult to determine if sustained repopulation of CD4^+^ T cells occurs in the oral mucosa during ART. Numerous longitudinal follow-up studies of HIV infected subjects using long-term highly active ART (HAART) have, however, shown that CD4^+^ T cells fail to continuously repopulate the mucosa even after 10 years of continuous HAART even though transient repopulation is apparent during therapy [28–32]. On the other hand, Guadalupe et al. [24] showed that there was a substantial delay in CD4^+^ T cell repopulation of the gut mucosal tissues during HAART. Longitudinal sampling of oral mucosal tissues during ART would be essential to address this question.

Interestingly, repopulating CD4^+^ T cells in the oral mucosa were found to harbor ~40% of Th17 cells suggesting that ART was capable of restoring oral mucosal immunity. Macal et al. [33] showed that effective repopulation of the gut lymphoid tissue was characterized by enhanced Th17 responses, whereas Klatt and Brenchley [34] suggested that effective repopulation of Th17 cells in the gastrointestinal mucosa was associated with better disease prognosis. Th17 cells play a key role in maintaining mucosal immune homeostasis and immunity and repopulation of these cells in the oral mucosa can potentially lead to better oral health during ART. Several studies [20, 35–38] have shown that the loss of Th17 cells was associated with translocation of microbial products and increased immune activation suggesting that repopulating Th17 cells may potentially limit translocation across oral mucosal surfaces. Additional studies are needed to clarify this question further.

Interestingly, ~2% of repopulating CD4^+^ T cells in the oral mucosa were found to be infected and carry viral DNA; there were ~4,000 copies of SIV DNA/10^5^ CD4^+^ T cells. Previous studies have shown that low levels of viral replication continued in the mucosal tissues of HIV infected patients and SIV infected macaques during highly suppressive ART [30, 31, 39–41]. These findings suggest that, like other mucosal tissues, the infected viral reservoir persists in the oral mucosa during ART. Our findings have significant implications for development of functional cure therapies that target the eradication of the oral HIV reservoir in patients undergoing highly suppressive HAART.

In conclusion, our studies provide novel insights into the dynamics of CD4^+^ T cell depletion and repopulation in the oral mucosa during chronic SIV infection and therapy. CD4^+^ T cells repopulating the oral mucosa harbor Th17 cells suggesting that ART can potentially restore immunity at oral mucosal surfaces. The persistence of low levels of infected viral reservoir, however, suggests that ART regimens likely need to be combined with other approaches to eradicate the oral viral reservoir leading to better and more effective immune reconstitution in the oral mucosa during HIV infection.
